# Effects of estetrol/drospirenone vs drospirenone-only on thrombin generation in women with polycystic ovary syndrome: a randomized, double-blind, controlled trial

**DOI:** 10.1016/j.rpth.2026.106897

**Published:** 2026-08-03

**Authors:** Panicha Chantrapanichkul, Paveenutch Wiwatboworn, Manee Rattanachaiyanont, Prasong Tanmahasamut, Thanyarat Wongwananuruk, Wichapanun Surasang, Theera Ruchutrakool, Yingyong Chinthammitr, Bundarika Suwanawiboon, Tarinee Rungjirajittranon

**Affiliations:** 1 Gynecologic Endocrinology Unit, Department of Obstetrics and Gynecology, Faculty of Medicine Siriraj Hospital, Mahidol University, Bangkok, Thailand; 2 Division of Hematology, Department of Medicine, Faculty of Medicine Siriraj Hospital, Mahidol University, Bangkok, Thailand

**Keywords:** drospirenone-only pill, estetrol/drospirenone, oral contraceptive, polycystic ovary syndrome, thrombin generation

## Abstract

**Background:**

Combined oral contraceptives are first-line treatment for polycystic ovary syndrome (PCOS); however, venous thromboembolism risk remains a concern. Estetrol/drospirenone (E4/DRSP) has a more favorable thrombin generation assay (TGA) profile than ethinyl estradiol–based combined oral contraceptives, but direct comparison with progestin-only pills is lacking.

**Objectives:**

This study aimed to compare the effects of E4/DRSP and DRSP-only on TGA parameters, hyperandrogenic symptoms, and vaginal bleeding patterns in women with PCOS.

**Methods:**

In a randomized, double-blind, controlled trial, women with PCOS aged 18 to 40 years at Siriraj Hospital, Thailand (May-December 2025) were assigned in a 1:1 ratio to E4/DRSP or DRSP-only for 12 weeks. The primary outcome was change in TGA parameters measured with and without thrombomodulin (TM). Secondary outcomes were vaginal bleeding patterns, modified Ferriman–Gallwey score, acne severity, and treatment satisfaction.

**Results:**

Both regimens increased endogenous thrombin potential and peak height on TGA, without TM (E4/DRSP: 1699 to 1888; DRSP-only: 1836 to 2085 nM·min; within-group *P* ≤ .017) and with TM (both *P* = .001), with no between-group differences (all *P* > .05). The endogenous thrombin potential-TM ratio rose in both groups, with no between-group difference (E4/DRSP: median +0.14; DRSP-only: +0.13; between-group *P* = .74). DRSP-only had fewer scheduled bleeding days across 3 cycles, whereas E4/DRSP reduced modified Ferriman–Gallwey score for hirsutism (*P* < .001). Waist-to-hip ratio, acne, and treatment satisfaction were comparable, with no serious adverse events.

**Conclusion:**

E4/DRSP and DRSP-only had comparable effects on thrombin generation and TM-mediated anticoagulation in women with PCOS. E4/DRSP reduced hirsutism, supporting its consideration as a treatment option for PCOS.

## Essentials

• Women with PCOS often use hormone pills that can elevate clot risk.
• In a double-blind trial, 66 women with PCOS took estetrol/drospirenone or drospirenone-only.
• Both pills raised blood-clotting activity to a similar degree, with no difference between them.
• Estetrol/drospirenone may be a suitable contraceptive option for women with PCOS.

## Graphical abstract

**Figure:**
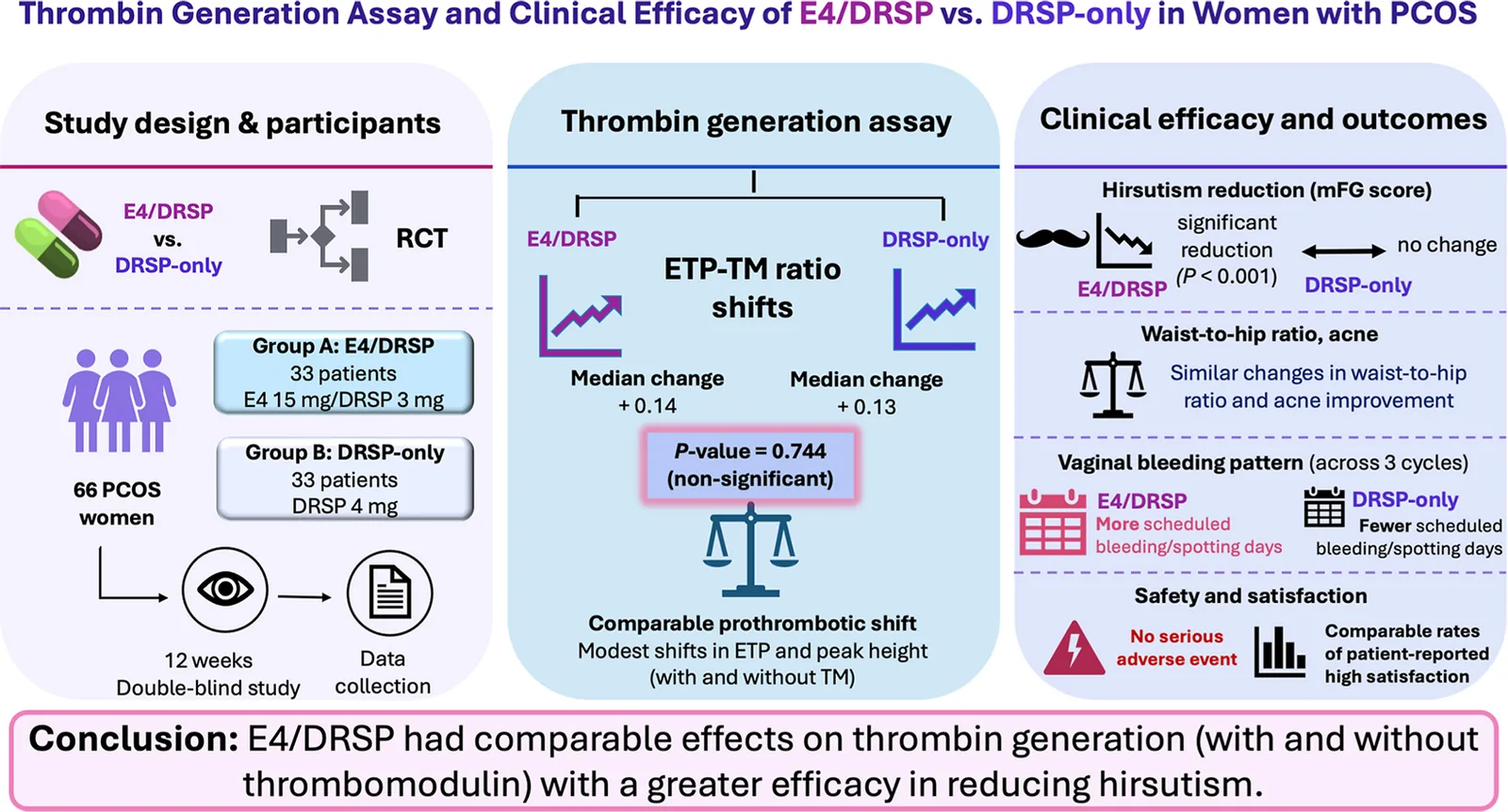

## 1 Introduction

Polycystic ovary syndrome (PCOS) is one of the most common hormonal and metabolic disorders affecting women of reproductive age [1]. Women with PCOS are at increased risk of comorbidities and long-term health consequences, including type 2 diabetes mellitus, hypertension, dyslipidemia, cardiovascular disease, and psychiatric disorders [[2], [3], [4]]. Beyond these metabolic disturbances, PCOS also carries an elevated risk of venous thromboembolism (VTE) [[5], [6], [7]].

Recent guidelines recommend combined oral contraceptives (COCs) as first-line treatment for women with PCOS [[8], [9], [10]]. Although COCs may raise the baseline risk of VTE, their benefits remain substantial [6,7]. COCs more effectively suppress hyperandrogenism, producing clinically meaningful improvements in acne and hirsutism [8]. In addition, COCs provide more predictable withdrawal bleeding and consistent endometrial protection against hyperplasia arising from chronic anovulation. By contrast, progestin-only therapy is often associated with irregular bleeding, less reliable cycle regulation, and limited effects on hyperandrogenism [11,12]. When the risks of COC therapy outweigh the benefits, progestin-only pills serve as a subsequent therapeutic option, primarily to ensure endometrial protection [[8], [9], [10]]. However, evidence supporting their efficacy in controlling hyperandrogenic manifestations remains limited [8,9].

Nevertheless, VTE remains the most clinically important concern when weighing the risks and benefits of COC therapy. COCs carry a higher risk of VTE than progestin-only pills, owing to the prothrombotic effects of the estrogen component [13]. Estrogen undergoes hepatic first-pass metabolism, upregulating the synthesis of procoagulant factors [14,15]. In addition, COC use induces acquired activated protein C resistance, further compounding thrombotic risk through reduced protein S levels and suppression of tissue factor pathway inhibitor [14,15].

Recently, a new estrogen class has emerged with the clinical introduction of estetrol (E4), a naturally occurring estrogen produced by the human fetal liver throughout gestation. Unlike other estrogenic compounds, E4 is not a substrate for cytochrome P450 enzymes and does not generate pharmacologically active metabolites [11,16]. E4 has been formulated as a novel estrogen component in COCs, available as a fixed-dose combination of E4 15 mg and drospirenone (DRSP) 3 mg (E4/DRSP) [17]. This combination has demonstrated therapeutic efficacy for PCOS-related symptoms with minimal impact on metabolic parameters, although direct evidence in this population remains limited [[16], [17], [18]]. With respect to coagulation, E4/DRSP has been associated with only a modest effect on coagulation profiles, substantially lower than that observed with ethinyl estradiol (EE)-containing COCs [19,20].

Given the concerns about VTE risk and the limited efficacy data for E4/DRSP in women with PCOS, we conducted a prospective, randomized controlled trial comparing E4/DRSP with DRSP-only pills. DRSP-only was selected as the comparator because it carries a comparatively lower thrombotic risk and has an established role in PCOS management. The primary outcome was the effect of each regimen on coagulation profiles, assessed by the thrombin generation assay (TGA). Secondary outcomes included vaginal bleeding patterns and patient-reported satisfaction. Anthropometric and clinical hyperandrogenism parameters were also evaluated, comprising body mass index (BMI), waist-to-hip ratio (WHR), modified Ferriman–Gallwey (mFG) score for hirsutism, and acne severity.

## 2 Methods

### 2.1 Study design and participants

This randomized, double-blind, controlled trial was conducted between May and December 2025 at the Gynecologic Endocrinology Unit, Siriraj Hospital, Mahidol University, Bangkok, Thailand. The study protocol was approved by the Siriraj Institutional Review Board and complied with the Declaration of Helsinki and international guidelines for human research protection. Written informed consent was obtained from all participants before enrollment.

Women diagnosed with PCOS according to the 2023 International Evidence-Based Guideline for the Assessment and Management of PCOS were eligible for inclusion [8]. Diagnosis required the presence of at least 2 of the following 3 criteria: (1) clinical or biochemical hyperandrogenism, (2) ovulatory dysfunction, and (3) polycystic ovarian morphology on ultrasonography or elevated anti-Müllerian hormone levels. Other causes of androgen excess were excluded [8].

Eligible participants were Thai (Southeast Asian) women aged 18 to 40 years with a BMI between 18.0 and 35.0 kg/m^2^. Exclusion criteria comprised contraindications to oral contraceptive use, prior use of estrogen-containing hormonal contraception within the preceding 3 months, and personal or family history of coagulation or bleeding disorders, known inherited thrombophilia, and current use of antiplatelet or anticoagulant therapy. Additional exclusions were diabetes mellitus, hypertension, cardiovascular disease, previous VTE, thyroid dysfunction, hyperprolactinemia, active smoking, and current or planned pregnancy.

### 2.2 Randomization and blinding

**Figure 1:**
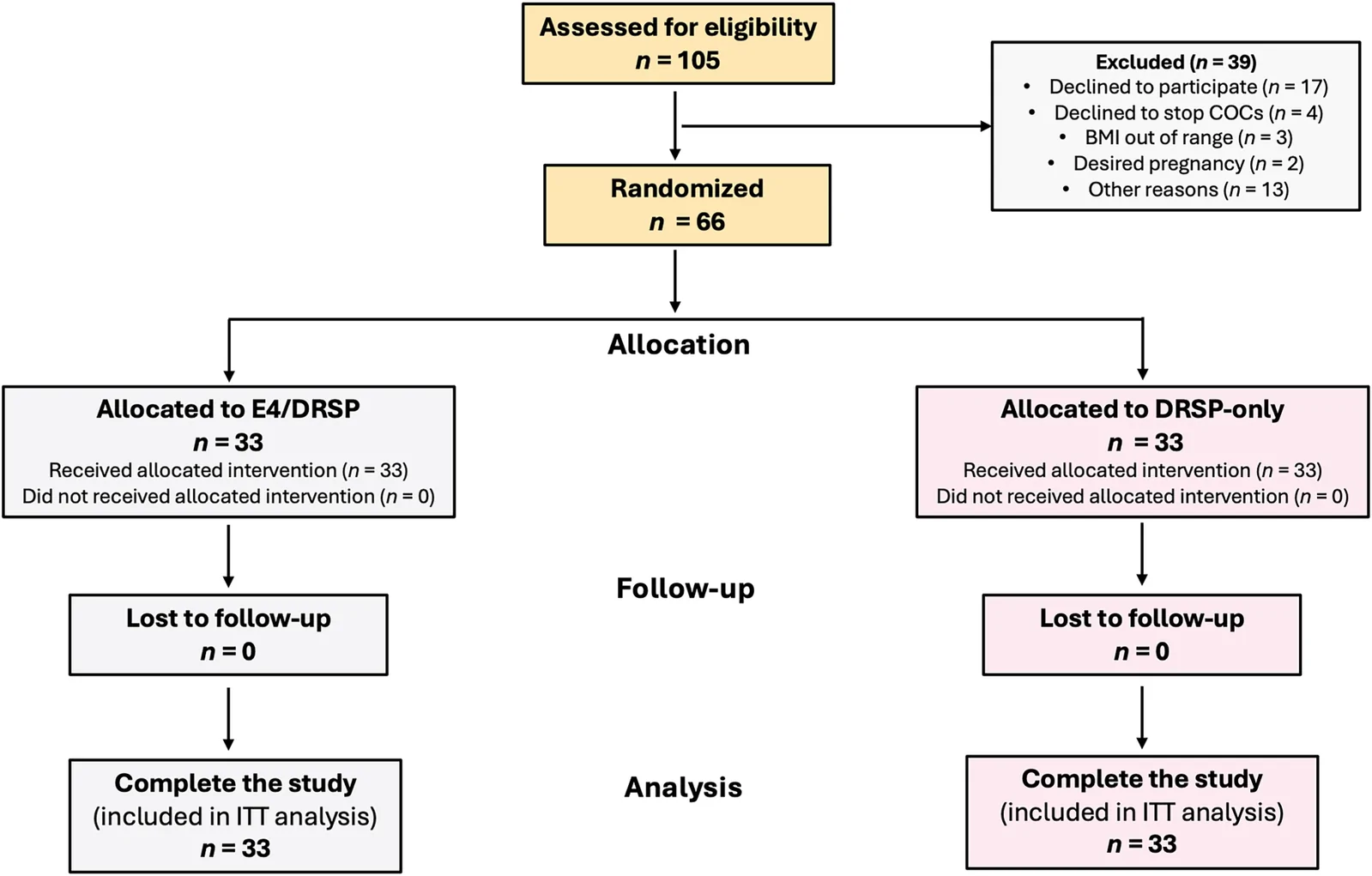
CONSORT flow diagram of study participants. BMI, body mass index; COCs, combined oral contraceptives; DRSP, drospirenone; E4, estetrol; ITT, intention-to-treat; *n*, number of patients.

Participants were randomly assigned in a 1:1 ratio to receive either E4/DRSP or DRSP-only, with 33 participants allocated to each group. Randomization was performed using a computer-generated block randomization scheme. Allocation concealment was ensured using sequentially numbered, sealed opaque envelopes prepared in advance and accessible only to the dispensing pharmacist (Figure 1).

To maintain double-blinding, all pills were individually overencapsulated by the Pharmacy Preparation Unit of Siriraj Hospital in a dedicated compounding facility. Active hormone tablets were placed in pink capsules and placebo pills in green capsules, with capsules identical in appearance across both treatment arms. Neither participants nor investigators were aware of treatment allocation throughout the study period.

### 2.3 Study interventions, blood sampling, and follow-up

Before initiation of the study medication, baseline physical examinations were performed, including blood pressure, BMI, WHR, mFG score, and acne severity assessment. In addition, all participants received medroxyprogesterone acetate 10 mg orally once daily for 10 days to induce withdrawal bleeding. This step standardized the menstrual cycle phase and minimized confounding from the irregular bleeding patterns inherent to PCOS. Baseline thrombophilia parameters were assessed before initiation of study medication. Protein C activity was measured by a chromogenic assay (Berichrom Protein C; Siemens Healthineers). Free protein S antigen levels were determined by an automated turbidimetric assay (Innovance Free PS Ag; Siemens Healthineers). Antithrombin activity was assessed with a chromogenic assay (Berichrom Antithrombin III [A]; Siemens Healthineers). All measurements were performed on days 2 to 7 after confirmation of induced withdrawal bleeding to exclude baseline inherited thrombophilic conditions. Pelvic ultrasonography was performed before blood sampling to confirm the absence of active ovulation [21]. Blood samples for TGA were then collected concurrently with thrombophilia testing.

After confirmation of withdrawal bleeding, participants initiated their assigned study medication 1 day after blood sampling was completed. Participants in the E4/DRSP group received a COC containing E4 15 mg and DRSP 3 mg, whereas those in the DRSP-only group received DRSP 4 mg. Both regimens followed a 24/4 dosing schedule (24 active hormone capsules followed by 4 placebo capsules) and were administered for 3 consecutive cycles (84 days). Participants were contacted by telephone or text message 4 weeks after treatment initiation to assess medication adherence and monitor for adverse events. They were also instructed to prospectively record any abnormal bleeding episodes throughout the study period.

If active pills were missed, participants were instructed to take the missed pill immediately upon recall and continue the remaining pills as scheduled. If 2 or more consecutive pills were missed, the most recently missed pill was to be taken immediately, and the remaining schedule continued. Barrier contraception was advised for 7 days if pregnancy prevention was desired. If pills were missed during the 3rd week of the active phase, participants were instructed to omit the placebo phase and start a new pack immediately. Missed placebo pills required no corrective action.

**Figure 2:**
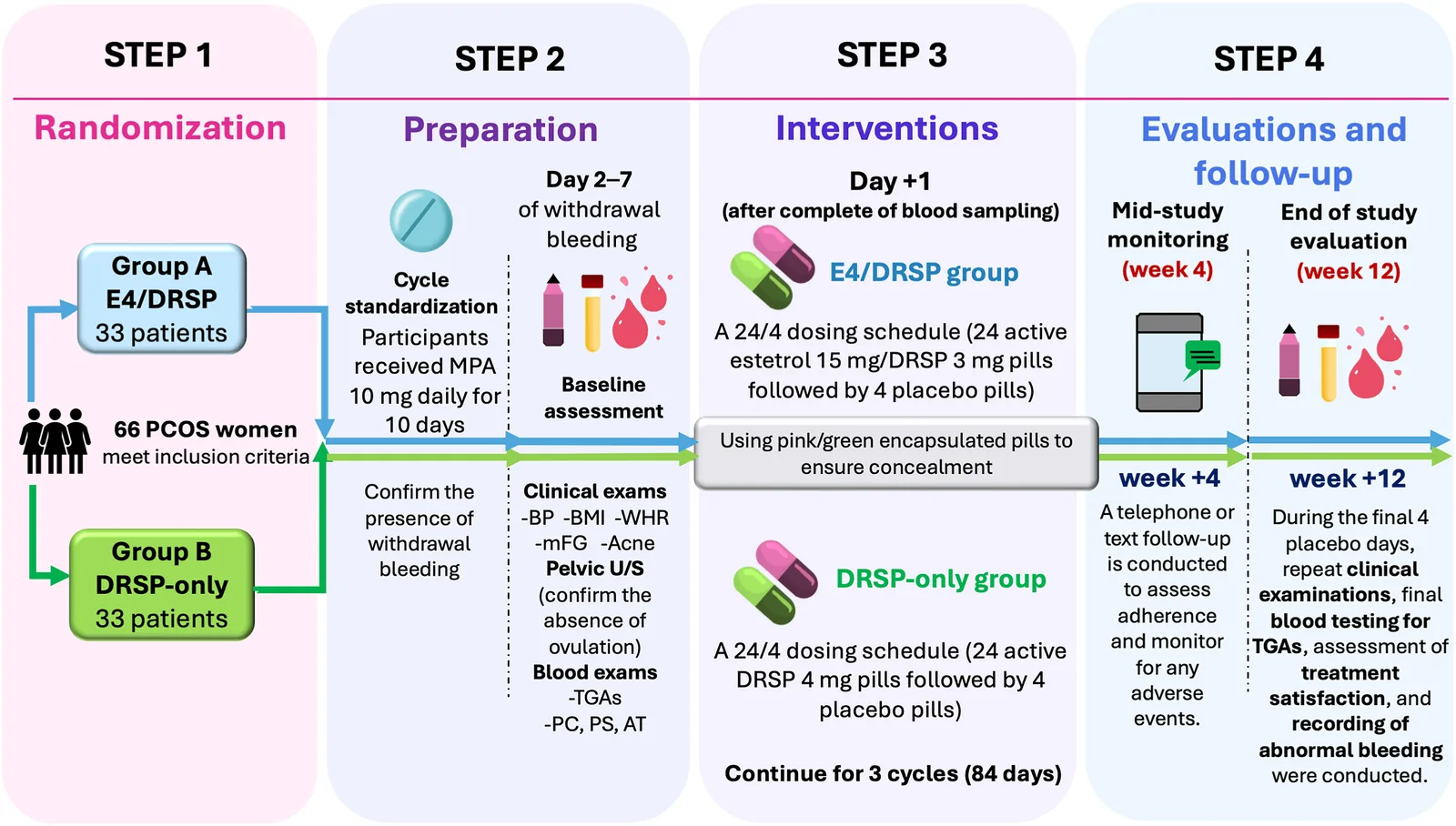
Schematic of study interventions and outcome assessments at baseline and after 12 weeks of treatment. AT, antithrombin; BMI, body mass index; BP, blood pressure; DRSP, drospirenone; E4, estetrol; mFG, modified Ferriman–Gallwey score; MPA, medroxyprogesterone acetate; PC, protein C; PCOS, polycystic ovary syndrome; PS, protein S; TGA, thrombin generation assay; U/S, ultrasonography; WHR, waist-to-hip ratio.

Participants returned for a follow-up visit after 12 weeks of treatment, during the final 4 placebo days. At this visit, blood pressure, BMI, WHR, mFG score, and acne severity were reassessed. Blood samples were collected to evaluate TGA after treatment. Treatment satisfaction was assessed, and participants were instructed to return their medication packs for adherence evaluation (Figure 2).

### 2.4 Study outcomes

The primary outcome was the change in TGA parameters after 12 weeks of treatment in women with PCOS, comparing the E4/DRSP and DRSP-only groups. A 12-week (3-cycle) treatment period was chosen because hormonal contraceptive–associated hemostatic changes are typically detectable within ∼8 to 12 weeks [13]. Secondary outcomes included between-group comparisons of vaginal bleeding patterns, anthropometric and clinical hyperandrogenism parameters (BMI, mFG score, WHR, and acne severity), and treatment satisfaction.

For thrombin generation measurements, blood was collected into 3.2% sodium citrate tubes and centrifuged at 3000*g* for 25 minutes to obtain platelet-poor plasma. Plasma was aliquoted and stored at −20 °C until analysis. Thrombin generation was assessed using a calibrated automated thrombogram (Thrombinoscope; Diagnostica Stago) according to established methods, with 5 pM tissue factor added to each well. To reduce interassay variability, all samples from each participant, including both time points, were analyzed in a single run [22]. Parameters included lag time, time to peak, peak thrombin, and endogenous thrombin potential (ETP) [22]. Assays were performed with and without thrombomodulin (TM) to assess sensitivity to the protein C pathway.

Bleeding patterns were recorded daily by participants using a diary card, with definitions adapted from Creinin et al. [23]. “Scheduled (withdrawal) bleeding” was defined as bleeding or spotting during the expected interval and lasting ≤7 days. “Unscheduled bleeding” was defined as any bleeding or spotting outside the expected interval and beyond the 7-day withdrawal window. “Spotting” was defined as minimal bleeding not requiring menstrual products.

Anthropometric and clinical hyperandrogenism parameters included WHR, defined as the ratio of waist circumference to hip circumference (waist/hip, cm/cm) [24]. Waist circumference was measured at the midpoint between the lowest rib and the iliac crest, in a standing position at end-expiration. Hip circumference was measured at the widest part of the buttocks [24]. Each measurement was performed twice, and the mean value was used. The mFG scoring system was used to assess hirsutism, with only terminal hairs >5 mm evaluated [25]. Acne severity was classified per the Thai Dermatological Society guideline as mild (<10 comedones or papules/pustules), moderate (>10 papules/pustules and/or <5 nodules), or severe (multiple papules/pustules or multiple nodules/cysts). The same physician assessed both hirsutism and acne throughout the study to minimize interobserver variability [26].

Patient-reported satisfaction was assessed using a 5-point Likert scale, ranging from “not at all satisfied” to “very satisfied” [27].

### 2.5 Sample size calculation

Based on previous studies, the relative change in peak thrombin after treatment with E4/DRSP was 17.3% ± 16.5% from baseline; a change of 5.0% ± 16.5% was assumed for the DRSP-only group [28]. A sample size of 29 participants per group was required to achieve 80% power at a two-sided alpha level of .05 to detect the expected between-group difference. Allowing for a 10% dropout rate, the sample size was increased to 33 participants per group, for a total of 66 participants.

### 2.6 Statistical analysis

Baseline characteristics were summarized using appropriate descriptive statistics. Continuous variables were expressed as mean (SD) or median (IQR), and categorical variables as number (%). Within-group comparisons used a paired *t*-test or Wilcoxon signed-rank test, as appropriate. Between-group comparisons used an independent *t*-test or Mann–Whitney *U* test, depending on data distribution. Bleeding days were summarized as median (IQR) and compared using the Mann–Whitney *U* test. Changes in BMI, WHR, and mFG score were analyzed using a paired *t*-test or Wilcoxon signed-rank test, as appropriate. Treatment satisfaction was reported as a number (%). A 2-sided *P* < .05 was considered statistically significant. All statistical analyses were performed using IBM SPSS Statistics, version 31 (IBM Corp).

## 3 Results

### 3.1 Baseline clinical and laboratory characteristics

A total of 66 PCOS participants were randomized into 2 groups: E4/DRSP (*n* = 33) and DRSP-only (*n* = 33). The mean (SD) age was 26.2 (4.8) and 25.8 (4.1) years, respectively. Anthropometric data showed mean (SD) body weights of 66.2 (13.9) and 67.5 (12.1) kg and mean (SD) BMIs of 25.7 (4.5) and 26.0 (4.6) kg/m^2^, respectively. Mean (SD) WHR was comparable between groups (0.81 [0.07] in both). Blood pressure was within the normal range in both groups. Mean (SD) systolic blood pressure was 118.7 (9.7) and 119.7 (12.2) mm Hg, and mean (SD) diastolic blood pressure was 69.5 (9.4) and 70.2 (11.1) mm Hg in the E4/DRSP and DRSP-only groups, respectively. Regarding hyperandrogenism, the median (IQR) mFG score was 1 (0-4) and 1 (0-2), and approximately half of participants in both groups had mild acne. Inherited thrombophilia testing showed normal protein C, protein S, and antithrombin activity in all participants. Table 1 summarizes the baseline characteristics and laboratory investigations. All 66 participants (33 per group) completed the 12-week follow-up, with no dropouts. Medication adherence, assessed by returned pill-pack counts, was 100% in both groups.

**Table 1:** Baseline clinical and laboratory characteristics.

Characteristics	E4/DRSP group (*n* = 33)	DRSP-only group (*n* = 33)
**Age**, mean (SD), y	26.2 (4.8)	25.8 (4.1)
**Body weight**, mean (SD), kg	66.2 (13.9)	67.5 (12.1)
BMI, mean (SD), kg/m^2^	25.7 (4.5)	26.0 (4.6)
**Waist-to-hip ratio**, mean (SD)	0.81 (0.07)	0.81 (0.07)
**SBP**, mean (SD), mm Hg	118.7 (9.7)	119.7 (12.2)
**DBP**, mean (SD), mm Hg	69.5 (9.4)	70.2 (11.1)
**mFG score,** median (IQR)	1 (0-4)	1 (0-2)
**Acne severity, *n* (%)**		
No acne	7 (21.2%)	10 (30.3%)
Mild	22 (66.7%)	17 (51.5%)
Moderate	2 (6.1%)	5 (15.2%)
Severe	2 (6.1%)	1 (3.0%)
Protein C activity, mean (SD), % (normal range 70%-140%)	110.1 (18.2)	113.8 (18.7)
Protein S activity, mean (SD), % (normal range 70%-130%)	93.7 (18.1)	95.4 (15.1)
Antithrombin activity, mean (SD), % (normal range 79.4%-112%)	106.5 (8.0)	105.1 (9.4)

Data are presented as mean (SD) for normally distributed variables and median (IQR) for nonnormally distributed variables. Categorical variables are presented as *n* (%).

BMI, body mass index; DBP, diastolic blood pressure; DRSP, drospirenone; E4, estetrol; mFG, modified Ferriman–Gallwey score; *n*, number of patients in group; SBP, systolic blood pressure.

### 3.2 Changes in TGA parameters without TM following E4/DRSP and DRSP-only treatment

To assess the effects of hormonal treatment on coagulation, TGA was performed both with and without TM. In the E4/DRSP group, median baseline values for TGA without TM were 2.7 minutes (2.5-3.1) for lag time, 1699.1 nM·min (1498.0-2001.0) for ETP, 278.5 nM (263.2-348.9) for peak height, and 5.7 minutes (5.4-6.5) for time to peak. After 12 weeks of treatment, ETP rose by a median of 282.1 nM·min (95% CI, 89.3-498.0) to 1888.1 nM·min (1694.4-2335.2; *P* = .01). Peak height and time to peak also shifted in a prothrombotic direction. Peak height increased by ∼72.6 nM (95% CI, 38.6-111.4) to 369.9 nM (330.2-413.0; *P* < .001), and time to peak shortened (*P* = .02). By contrast, lag time was comparable before and after treatment (*P* = .068; Table 2).

**Table 2:** Thrombin generation parameters at baseline and after 12 weeks in the E4/DRSP and DRSP-only groups.

Variable	Group	*n*	Baseline median (IQR)	12 Weeks median (IQR)	Median change (12 weeks − baseline)	95% CI	*P* value^a^	*P* value^b^
***Without TM***								
Lag time, min	E4/DRSP	33	2.7 (2.5-3.1)	2.7 (2.3-3.0)	−0.3	−0.3 to 0.0	0.068	0.378
	DRSP-only	33	3.0 (2.7-3.2)	2.7 (2.3-3.0)	−0.3	−0.5 to 0.0	0.04	
ETP, nM·min	E4/DRSP	33	1699.1 (1498.0-2001.0)	1888.1 (1694.4-2335.2)	282.1	89.3 to 498.0	0.011	0.401
	DRSP-only	33	1836.2 (1677.2-2014.3)	2084.5 (1692.1-2320.2)	76.8	−5.1 to 253.3	0.02	
Peak height, nM	E4/DRSP	33	278.5 (263.2-348.9)	369.9 (330.2-413.0)	72.6	38.6 to 111.4	< 0.001	0.803
	DRSP-only	33	296.2 (264.4-348.1)	369.9 (340.2-428.3)	74.2	16.4 to 95.1	< 0.001	
Time to peak, min	E4/DRSP	33	5.7 (5.4-6.5)	5.3 (4.7-5.8)	−0.5	−1.2 to −0.1	0.02	0.314
	DRSP-only	33	5.9 (5.7-7.0)	5.3 (4.6-6.3)	−0.8	−1.7 to −0.2	0.003	
***With TM***								
Lag time, min	E4/DRSP	33	2.7 (2.5-3.0)	2.7 (2.3-3.0)	−0.2	−0.3 to 0.0	0.060	0.995
	DRSP-only	33	2.7 (2.4-3.0)	2.5 (2.3-3.1)	−0.2	−0.3 to 0.0	0.159	
ETP, nM·min	E4/DRSP	33	1190.7 (972.1-1445.1)	1519.7 (1357.9-1848.9)	296.8	181.8 to 515.8	0.001	0.763
	DRSP-only	33	1202.6 (1021.2-1449.0)	1586.8 (1426.3-1886.4)	370.7	224.2 to 469.1	< 0.001	
Peak height, nM	E4/DRSP	33	246.7 (216.4-308.9)	325.1 (286.7-409.1)	92.6	26.4 to 136.2	0.001	0.803
	DRSP-only	33	246.9 (198.5-298.8)	327.8 (296.5-408.5)	93.3	51.2 to 151.4	< 0.001	
Time to peak, min	E4/DRSP	33	5.3 (5.1-5.7)	5.0 (4.7-5.4)	−0.3	−0.7 to 0.0	0.057	0.710
	DRSP-only	33	5.3 (5.1-5.7)	4.8 (4.5-5.8)	−0.4	−0.8 to 0.0	0.080	
***ETP-TM ratio (ETP with TM/ETP without TM)***								
ETP-TM ratio	E4/DRSP	33	0.72 (0.63-0.78)	0.86 (0.76-0.89)	0.14	0.03 to 0.20	0.002	0.744
	DRSP-only	33	0.66 (0.55-0.79)	0.83 (0.68-0.88)	0.13	0.08 to 0.15	0.001	

DRSP, drospirenone; E4, estetrol; ETP, endogenous thrombin potential; *n*, number of patients in group; TM, thrombomodulin.

a *P* value for the within-group change from baseline to 12 weeks calculated using the Wilcoxon signed-rank test.

b *P* value for the between-group change from baseline to 12 weeks calculated using the Mann–Whitney *U* test.

In the DRSP-only group, median baseline values were 3.0 minutes (2.7-3.2) for lag time, 1836.2 nM·min (1677.2-2014.3) for ETP, 296.2 nM (264.4-348.1) for peak height, and 5.9 minutes (5.7-7.0) for time to peak. After 12 weeks of treatment, ETP rose by a median of 76.8 nM·min (95% CI, −5.1 to 253.3) to 2084.5 nM·min (1692.1-2320.2; *P* = .017). Peak height also rose by 74.2 nM (95% CI, 16.4-95.1) to 369.9 nM (340.2-428.3; *P* < .001), and time to peak shortened by a median of −0.8 minutes (95% CI, −1.7 to −0.2; *P* = .003). Lag time showed a borderline decrease (*P* = .039; Table 2).

Between-group comparisons showed no statistically significant differences in TGA parameters without TM between the E4/DRSP and DRSP-only groups (all *P* > .05; Table 2).

### 3.3 Changes in TGA parameters with TM following E4/DRSP and DRSP-only treatment

TGA was also performed with TM added to better characterize the effect of natural anticoagulant proteins, particularly protein C, in these participants. In the E4/DRSP group, baseline median values were 2.7 minutes (2.5-3.0) for lag time, 1190.7 nM·min (972.1-1445.1) for ETP, 246.7 nM (216.4-308.9) for peak height, and 5.3 minutes (5.1-5.7) for time to peak. After 12 weeks of treatment, ETP rose by a median of 296.8 nM·min (95% CI, 181.8-515.8) to 1519.7 nM·min (1357.9-1848.9; *P* = .001). Peak height also rose by 92.6 nM (95% CI, 26.4-136.2) to 325.1 nM (286.7-409.1; *P* = .001). Lag time and time to peak did not change appreciably (*P* = .06 and *P* = .06, respectively; Table 2).

In the DRSP-only group, baseline median values were 2.7 minutes (2.4-3.0) for lag time, 1202.6 nM·min (1021.2-1449.0) for ETP, 246.9 nM (198.5-298.8) for peak height, and 5.3 minutes (5.1-5.7) for time to peak. After 12 weeks of treatment, ETP rose by a median of 370.7 nM·min (95% CI, 224.2-469.1) to 1586.8 nM·min (1426.3-1886.4; *P* < .001). Peak height also rose by 93.3 nM (95% CI, 51.2-151.4) to 327.8 nM (296.5-408.5; *P* < .001). Lag time and time to peak did not change appreciably (*P* = .16 and *P* = .08, respectively; Table 2).

Between-group comparisons again showed no statistically significant differences in TGA parameters with TM between the E4/DRSP and DRSP-only groups (all *P* > .05; Table 2).

### 3.4 ETP-TM ratio following E4/DRSP and DRSP-only treatment

In the E4/DRSP group, the median ETP-TM ratio rose from 0.72 (0.63-0.78) at baseline to 0.86 (0.76-0.89) at 12 weeks, with a median change of 0.14 (95% CI, 0.03-0.20; *P* = .002). Similarly, in the DRSP-only group, the median ETP-TM ratio rose from 0.66 (0.55-0.79) to 0.83 (0.68-0.88), with a median change of 0.13 (95% CI, 0.08-0.15; *P* = .001; Table 2).

**FigurE 3:**
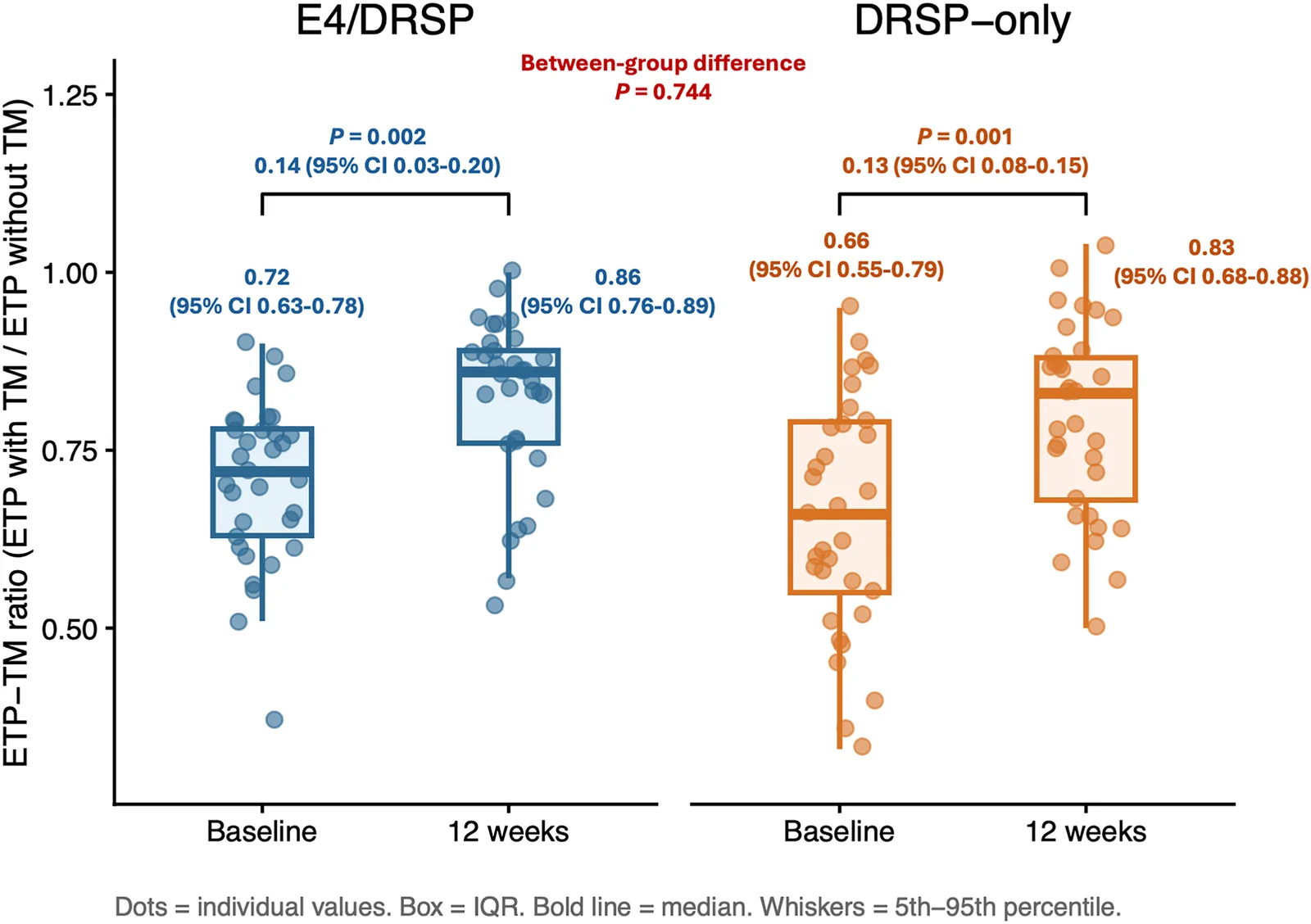
Changes in ETP-TM ratio from baseline to 12 weeks in the E4/DRSP and DRSP-only groups. DRSP, drospirenone; E4, estetrol; ETP, endogenous thrombin potential; TM, thrombomodulin.

The magnitude of change in ETP-TM ratio did not differ between the E4/DRSP and DRSP-only groups (*P* = .74). This finding suggests that the prothrombotic effect on coagulation balance was comparable regardless of the estrogen component used (Table 2; Figure 3).

### 3.5 Vaginal bleeding patterns following E4/DRSP and DRSP-only treatment

Vaginal bleeding patterns, including scheduled and unscheduled bleeding or spotting days per cycle, are summarized in Supplementary Table 1. Participants receiving DRSP-only had fewer scheduled bleeding or spotting days than those receiving E4/DRSP across all 3 cycles (cycle 1: *P* = .01; cycle 2: *P* = .002; cycle 3: *P* < .001). In both groups, the number of scheduled bleeding or spotting days tended to decrease over successive cycles. For unscheduled bleeding or spotting, a statistically significant between-group difference was observed in cycle 1 (*P* = .02), whereas no statistically significant differences were detected in cycles 2 and 3 (*P* = .939 and *P* = .336, respectively).

### 3.6 Changes in anthropometric and clinical hyperandrogenism parameters following E4/DRSP and DRSP-only treatment

As shown in Supplementary Table 2, WHR did not change appreciably from baseline to 12 weeks in either treatment group (both *P* > .05). For hirsutism, the mFG score did not change in the DRSP-only group (*P* = .336). However, a statistically significant reduction was observed in the E4/DRSP group, from a median of 1 (0-4) at baseline to 0.5 (0-3) at 12 weeks (*P* < .001). Acne outcomes at 12 weeks are summarized in Supplementary Table 3, with comparable proportions of participants showing no change, ≥1-grade improvement, or worsening between groups (*P* = .827).

### 3.7 Treatment satisfaction in the E4/DRSP and DRSP-only groups

At 12 weeks, the rate of patient-reported high satisfaction was comparable between groups (E4/DRSP: 93.9% vs DRSP-only: 84.8%; *P* = .28). The proportion of participants expressing a desire to continue treatment was also similar (E4/DRSP: 90.9% vs DRSP-only: 75.8%; *P* = .099).

### 3.8 Adverse events in the E4/DRSP and DRSP-only groups

Adverse events are summarized in Supplementary Table 3. The incidence of adverse events, including nausea, acne, breast engorgement, mood changes, and headache, did not differ between the E4/DRSP and DRSP-only groups (all *P* > .05). No serious adverse events or treatment discontinuations due to adverse events were reported in either group.

## 4 Discussion

Our study demonstrated prothrombotic effects of both E4/DRSP and DRSP-only on TGA parameters. TGA is a sensitive functional assay reflecting the overall balance between procoagulant and anticoagulant pathways and is considered the primary mechanism by which hormonal contraceptives influence coagulation. Few previous studies have directly compared coagulation effects between these 2 regimens in a PCOS population. Both treatments were associated with prothrombotic shifts in TGA profiles after 12 weeks, with no statistically significant difference between groups. We also evaluated the efficacy of both treatments in controlling PCOS symptoms, as well as treatment satisfaction and adverse events.

Notably, baseline TGA parameters in our cohort revealed a more prothrombotic phenotype in women with PCOS, with elevated ETP values exceeding those reported in the general population [29]. This pattern is consistent with the known association between PCOS and elevated VTE risk, attributable to chronic inflammation, increased endogenous estrogen production, and metabolic risk factors including obesity [[5], [6], [7]]. In line with this, participants in our study had body weights in the overweight to obese range based on Asian BMI cutoffs (overweight ≥ 23 kg/m^2^; obesity ≥ 27.5 kg/m^2^) [30]. All participants also received medroxyprogesterone acetate 10 mg daily for 10 days before study initiation to standardize the menstrual cycle phase. This pretreatment may have contributed to the elevated baseline TGA parameters in our cohort, in addition to the prothrombotic phenotype inherent to PCOS itself. These baseline values should therefore be interpreted in the context of prior progestin exposure [31]. Importantly, no participant had evidence of inherited thrombophilia, including protein C, protein S, or antithrombin deficiencies, which are the predominant forms of inherited thrombophilia in Thai and East Asian populations [32].

Previous studies have demonstrated a lower prothrombotic profile with E4/DRSP on TGA, consistent with the lower thrombotic risk associated with natural estrogens compared with EE-based COCs [33]. Douxfils et al. [19] demonstrated a lower effect of E4/DRSP on hemostatic parameters than EE/DRSP or EE/levonorgestrel. Similarly, Morimont et al. [28] reported a more neutral hemostatic profile with E4/DRSP.

DRSP-only, as a type of progestin-only pill, has been associated with a lower risk of thrombosis than combined COCs [13,34]. Progestin subdermal implants and levonorgestrel-releasing intrauterine devices have also been reported to have no effect on activated protein C resistance [35,36]. To date, no studies have directly evaluated changes in thrombin generation following the use of DRSP-only.

Our study revealed that most TGA parameters, particularly ETP and peak height, shifted in a prothrombotic direction, and this effect persisted after the addition of TM. These findings provide novel evidence of a prothrombotic shift in TGA parameters with DRSP-only. We hypothesize that this finding may be partly explained by the baseline hyperinflammatory state inherent to PCOS. This state may attenuate protein C pathway function and amplify the coagulation response to hormonal treatment beyond what would be expected in healthy women. Furthermore, TGA with TM may have helped detect subtle hemostatic changes that would remain undetected by conventional assays measuring only individual procoagulant and anticoagulant protein levels [37]. Future studies in healthy women without PCOS are warranted to confirm whether the observed prothrombotic TGA changes represent a true drug effect of DRSP-only or are specific to the PCOS phenotype. Participants in the E4/DRSP group also showed TGA parameter changes in the same prothrombotic direction, with no statistically significant difference between the 2 groups.

To further evaluate the overall impact of hormonal treatment on coagulation balance, the ETP-TM ratio was assessed. This ratio reflects the sensitivity of the coagulation system to TM-mediated anticoagulation, which encompasses the protein C pathway [22]. Both groups demonstrated a statistically significant rise in ETP-TM ratio following treatment. These findings indicate that both E4/DRSP and DRSP-only reduced the sensitivity of the coagulation system to TM-mediated anticoagulation, consistent with an effect on the protein C anticoagulant pathway. The magnitude of this effect did not differ between the 2 groups. Although the observed increases in ETP-TM were statistically significant, the changes were modest, with a median increase of 0.13 to 0.14 in both groups. Direct numerical comparison with activated protein C resistance measures from other studies is limited by methodological differences. Nevertheless, the observed changes appear substantially lower than those reported with EE-based COCs [19], suggesting that the overall impact on coagulation balance for both regimens may be comparatively limited. However, these TGA parameters are surrogate markers of hemostatic activation and do not directly quantify clinical thrombotic risk; the prothrombotic shifts observed here should not be interpreted as an absolute increase in venous thromboembolic events.

We also evaluated the effect of both regimens on clinical hyperandrogenism. Acne improvement did not differ between groups, which may partly reflect the relatively short follow-up for this outcome. For hirsutism, a statistically significant reduction in mFG score was observed in the E4/DRSP group, whereas no change was detected in the DRSP-only group. This difference may be attributable to E4’s ability to increase hepatic sex hormone–binding globulin production, thereby reducing free testosterone activity [38]. However, the clinical relevance of this finding is uncertain because of the low baseline mFG scores in our cohort, which may have limited the detection of meaningful change. Studies with higher baseline hirsutism scores and longer treatment duration are warranted. For vaginal bleeding outcomes, DRSP-only was associated with fewer scheduled bleeding days than E4/DRSP, likely reflecting the absence of estrogen-induced withdrawal bleeding.

Our findings add to the limited evidence on changes in thrombin generation between E4/DRSP and DRSP-only in women with PCOS. The comparable prothrombotic TGA profiles between groups may inform clinical decision-making when E4/DRSP is considered as an alternative to progestin-only pills for managing hyperandrogenic symptoms. Furthermore, the modest rise in ETP-TM ratio observed in both groups may suggest a comparatively limited attenuation of TM-mediated anticoagulation relative to EE-based COCs, although direct comparison is warranted.

Several limitations should be acknowledged. First, the treatment duration was limited to 12 weeks. This duration may not be sufficient to capture long-term hemostatic changes or clinically meaningful changes in parameters such as clinical thrombosis, acne, and hirsutism. A longer exposure might also reveal between-group differences in certain outcomes that were not apparent at 12 weeks. Second, coagulation factor levels were not measured, precluding a mechanistic explanation of the observed changes in protein C pathway sensitivity as reflected by the ETP-TM ratio. Third, the use of medroxyprogesterone acetate before study initiation may have partially influenced baseline TGA parameters, which should be considered when interpreting the elevated baseline values. Fourth, our study population consisted exclusively of Southeast Asian women with PCOS. Therefore, the findings may not be generalizable to populations with different ethnic backgrounds or thrombophilia risk profiles, particularly those with a higher prevalence of factor V Leiden mutation, such as Caucasian women. Fifth, baseline mFG scores in our cohort were relatively low, which may have limited the ability to detect clinically meaningful reductions in hirsutism. Sixth, the effects of both treatments were derived from women with PCOS, which requires confirmation in healthy women. Finally, because no participant had inherited thrombophilia, we were unable to examine whether the hemostatic response to treatment is modified in patients with protein C, protein S, or antithrombin deficiency; this warrants dedicated study in higher-risk populations.

## 5 Conclusion

Our study demonstrated comparable TGA profiles following E4/DRSP and DRSP-only treatment in women with PCOS. This finding persisted with the addition of TM, reflecting a comparable reduction in sensitivity to TM-mediated anticoagulation between groups. Furthermore, E4/DRSP appeared to offer greater efficacy in controlling hyperandrogenic symptoms, particularly hirsutism, without notable adverse events. These findings suggest that E4/DRSP may be a promising option for women with PCOS who require management of hyperandrogenic symptoms. Further studies with clinical endpoints and longer follow-up are warranted to confirm these findings.

## Supplementary material

Supplementary Table S1 Number of scheduled and unscheduled vaginal bleeding or spotting days per cycle in the E4/DRSP and DRSP-only groups

Supplementary Table S2 Comparison of anthropometric and clinical hyperandrogenism parameters at baseline and after 12 weeks in the E4/DRSP and DRSP-only groups

Supplementary Table S3 Adverse events in the E4/DRSP and DRSP-only groups
