# Supplementary material for: Effects of estetrol/drospirenone vs drospirenone-only on thrombin generation in women with polycystic ovary syndrome: a randomized, double-blind, controlled trial
Source: Res Pract Thromb Haemost. 2026 Aug 3;10(5):106897. doi: 10.1016/j.rpth.2026.106897 (PMC13521139; doi:10.1016/j.rpth.2026.106897)
Supplement: Supplementary file 1 [file mmc1.docx]

**SUPPLEMENTARY TABLE S1** Number of scheduled and unscheduled vaginal bleeding or spotting days per cycle in the E4/DRSP and DRSP-only groups

| **Variable** | **E4/DRSP group *n* = 33** | **DRSP-only group *n* = 33** | ***P* value** |
| --- | --- | --- | --- |
| Scheduled bleeding/spotting | | | |
| Cycle 1, days | 4.0 (3.0–5.0) | 2.0 (0.0–4.5) | 0.013 |
| Cycle 2, days | 4.0 (2.0–5.0) | 1.0 (0.0–3.5) | 0.002 |
| Cycle 3, days | 4.0 (3.0–5.5) | 0.0 (0.0–3.0) | < 0.001 |
| Unscheduled bleeding/spotting | | | |
| Cycle 1, days | 0.0 (0.0–0.5) | 0.0 (0.0–4.5) | 0.020 |
| Cycle 2, days | 0.0 (0.0–2.0) | 0.0 (0.0–1.5) | 0.939 |
| Cycle 3, days | 0.0 (0.0–1.0) | 0.0 (0.0–0.0) | 0.336 |

Data are presented as median (IQR). *P* values were calculated using the Mann–Whitney *U* test.
**Abbreviations:** DRSP, drospirenone; E4, estetrol; *n*, number of patients in group.
