# Supplementary material for: Effects of estetrol/drospirenone vs drospirenone-only on thrombin generation in women with polycystic ovary syndrome: a randomized, double-blind, controlled trial
Source: Res Pract Thromb Haemost. 2026 Aug 3;10(5):106897. doi: 10.1016/j.rpth.2026.106897 (PMC13521139; doi:10.1016/j.rpth.2026.106897)
Supplement: Supplementary file 2 [file mmc2.docx]

**SUPPLEMENTARY TABLE S2** Comparison of anthropometric and clinical hyperandrogenism parameters at baseline and after 12 weeks in the E4/DRSP and DRSP-only groups

| **Variable** | **E4/DRSP group *n* = 33** | **DRSP-only group *n* = 33** |
| --- | --- | --- |
| Body weight | | |
| Baseline, mean (SD), kg | 66.2 (13.9) | 67.5 (12.1) |
| At 12 weeks, mean (SD), kg | 65.6 (14.2) | 67.5 (12.8) |
| ***P* value** | 0.224 | 0.922 |
| Waist-to-hip ratio | | |
| Baseline, mean (SD) | 0.81 (0.07) | 0.81 (0.07) |
| At 12 weeks, mean (SD) | 0.81 (0.06) | 0.81 (0.07) |
| ***P* value** | 0.581 | 0.537 |
| mFG scoring system | | |
| Baseline, median (IQR) | 1 (0–4) | 1 (0–2) |
| At 12 weeks, median (IQR) | 0.5 (0–3) | 0 (0–2) |
| ***P* value** | < 0.001 | 0.336 |
| **Acne improvement at 12 weeks post-treatment** | | |
| No change, *n* (%) | 22 (66.7%) | 20 (60.6%) |
| ≥ 1-grade improvement, *n* (%) | 6 (18.2%) | 8 (24.2%) |
| Worsened, *n* (%) | 5 (15.2%) | 5 (15.2%) |

Within-group changes were tested using paired *t* test or Wilcoxon signed-rank test, as appropriate; *P* values shown.
**Abbreviations:** DRSP, drospirenone; E4, estetrol; mFG, modified Ferriman–Gallwey score; *n*, number of patients in group; *n*, number of patients with event.
