# Supplementary material for: Effects of estetrol/drospirenone vs drospirenone-only on thrombin generation in women with polycystic ovary syndrome: a randomized, double-blind, controlled trial
Source: Res Pract Thromb Haemost. 2026 Aug 3;10(5):106897. doi: 10.1016/j.rpth.2026.106897 (PMC13521139; doi:10.1016/j.rpth.2026.106897)
Supplement: Supplementary file 3 [file mmc3.docx]

**SUPPLEMENTARY TABLE S3** Adverse events in the E4/DRSP and DRSP-only groups

| **Adverse events** | **E4/DRSP group *n* = 33** | **DRSP-only group *n* = 33** | ***P* value** |
| --- | --- | --- | --- |
| Nausea, *n* (%) | 2 (6.1%) | 4 (12.1%) | 0.672 |
| Acne, *n* (%)* | 5 (15.2%) | 8 (24.2%) | 0.353 |
| Breast engorgement, *n* (%) | 8 (24.2%) | 9 (27.3%) | 0.778 |
| Mood changes, *n* (%) | 12 (36.4%) | 12 (36.4%) | 1.000 |
| Headache, *n* (%) | 2 (6.1%) | 3 (9.1%) | 1.000 |

Between-group comparisons used the Fisher exact test; *P* values shown.
* “Acne” refers to new-onset or worsening acne reported as an adverse event during the treatment period.
**Abbreviations:** DRSP, drospirenone; E4, estetrol; *n*, number of patients in group; *n*, number of patients with event.
